# Reaction-dependent optical behavior and theoretical perspectives of colloidal ZnSe quantum dots

**DOI:** 10.1038/s41598-024-64995-5

**Published:** 2024-06-17

**Authors:** Duy Hoang Nguyen, Sung Hun Kim, Joon Sue Lee, Dong Su Lee, Hong Seok Lee

**Affiliations:** 1 Department of Physics, Research Institute Physics and Chemistry, Jeonbuk National University, Jeonju, 54896 Republic of Korea; 2 Department of Physics and Astronomy, University of Tennessee, Knoxville, TN 37996 USA; 3 Institute of Advanced Composite Materials, Korea Institute of Science and Technology (KIST), Wanju, 55324 Republic of Korea

**Keywords:** Quantum dots, ZnSe, Reaction times, Optical properties, Morphological properties, Materials science, Nanoscale materials, Quantum dots

## Abstract

Colloidal quantum dots (QDs) are attracting research interest because of their unique optical properties that result from the quantum confinement effect. ZnSe QDs, which are II–VI semiconductors, offer a wide direct bandgap (2.7 eV), making them promising for applications such as light-emitting diodes, photodetectors, and biomedical labeling. In the present work, colloidal ZnSe (QDs) were synthesized by the hot-injection method with a Zn:Se ratio of 1:1. The optical properties of ZnSe QDs obtained at different reaction times were investigated by spectrophotometric UV–vis absorption and emission measurements. The as-synthesized ZnSe QDs exhibit blue excitonic emission, and no defect emission was detected. Transmission electron micrographs indicated that the QDs have a spherical morphology with dimensions ranging from 3.69 to 4.53 nm. In particular, the Brus model was applied to demonstrate a correlation between the QD sizes and the optical bandgaps obtained from Tauc plots.

## Introduction

Colloidal quantum dots (QDs) are nanoparticles with physical dimensions of a few nanometers. When QDs are smaller than the Bohr radius of the material, the quantum confinement effect occurs, leading to a blue-shift to higher energy^1^. The quantum size effect imparts colloidal QDs with a unique property: their optical and electrical properties can be tuned on the basis of their size. QDs have thus been intensively studied over the past three decades due to their unique optical and electrical properties and wide range of technological applications in catalysts, solar cells, biosensors, light-emitting diodes, and displays^2^.

Group II–VI semiconductor QDs, which are commonly Cd-based compounds, have been extensively studied for their fundamental properties and technological applications^3,4^. However, Cd is toxic, which limits the practical applications these QDs and raises concerns about their potential threats to the environment and human health^3,5^. Therefore, there is increasing demand for the development of Cd-free QDs^6^. ZnSe, an important II–VI semiconductor material with a wide bandgap of 2.7 eV and low toxicity, shows promise as a replacement for Cd-based QDs^7^. Because of their uncommon UV–blue luminescence properties, ZnSe QDs have strong potential for use in low-voltage electroluminescent devices and blue diode lasers^8,9^.

The wide bandgap of ZnSe QDs also enables them to be used as an inorganic passivation shell in various core/shell structure materials or doped with transition metal ions, enabling tunable emission spanning the range of visible wavelengths^10,11^. Since optical properties strongly depend on size, structure, and composition, it is crucial to understand synthesis conditions for the production of high-quality ZnSe QDs. So far, monodispersed ZnSe nanocrystals have been successfully prepared by various solution-based methods, including hot-injection and heating methods^12^. In the hot-injection method, a reagent is injected into a hot reaction solution at room temperature, causing burst homogeneous nucleation, followed by slow growth of the QDs^6^. Moreover, other studies have been reported that Zn/Se ratios that differ from 1:1 do not result in improved optical properties of ZnSe QDs^13,14^.

In the present study, the hot-injection technique was used to synthesize high-quality ZnSe QDs with a Zn/Se ratio of 1:1. The optical and morphological properties of the prepared ZnSe QDs were investigated by UV–vis absorption spectroscopy, photoluminescence (PL) spectroscopy, and transmission electron microscopy (TEM).

## Results

### Synthesis mechanism of colloidal ZnSe QDs

**Figure 1:**
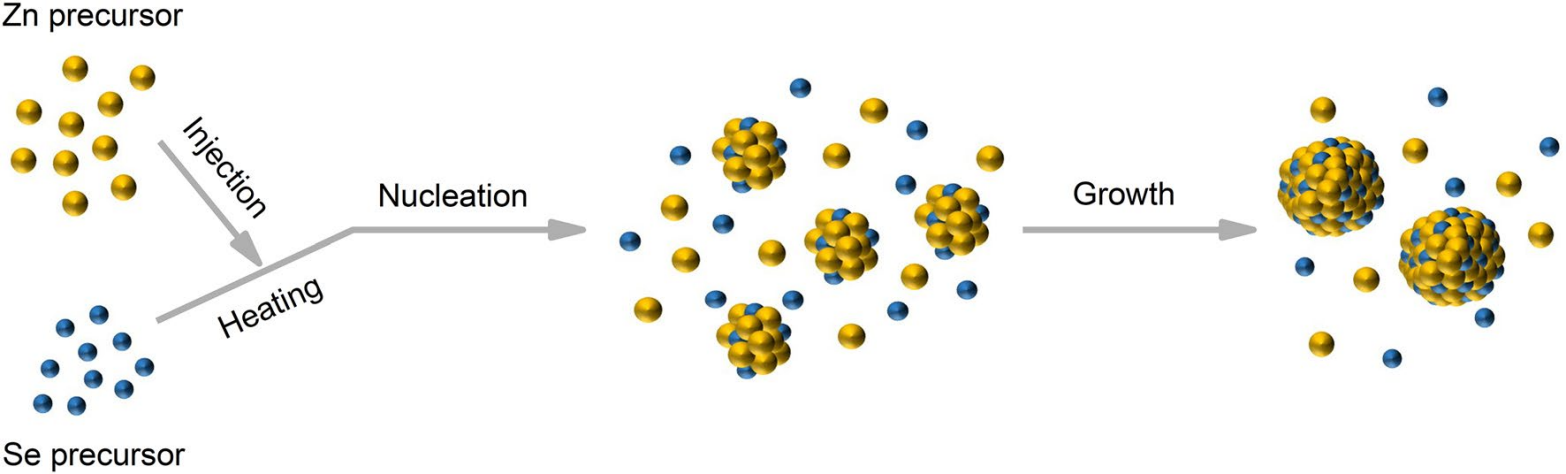
Schematic of the nucleation and growth process of the ZnSe QDs synthesized by the hot-injection method.

Figure 1 shows the synthesis scheme of the ZnSe QDs obtained by the hot-injection technique. This reaction scheme involves rapidly injecting a Se precursor into a hot Zn precursor, which leads to temporary separation nucleation and growth of ZnSe QDs^14^. A balance between nucleation and growth is necessary to control the QD size and size distribution^15–17^, which requires the synthesis to start with burst nucleation^17^ followed by slow growth to achieve precise size control^18^. Therefore, the reaction temperature and the reactivity of the monomers play important roles in the nucleation and growth of ZnSe QDs. High injection and growth temperatures increase the reaction rate. In addition, the selected precursor, zinc stearate (ZnSt_2_), is a long-alkyl-chain zinc carboxylate with a strong steric effect, which typically results in a low growth rate^15,19^. The ZnSt_2_ not only serves as a zinc source but also provides capping ligands^18,20^. However, to achieve the desired balance between nucleation and growth, we selected octadecylamine (ODA) as the reagent to activate the zinc carboxylate precursors^18^.

### Absorption and photoluminescence analysis

**Figure 2:**
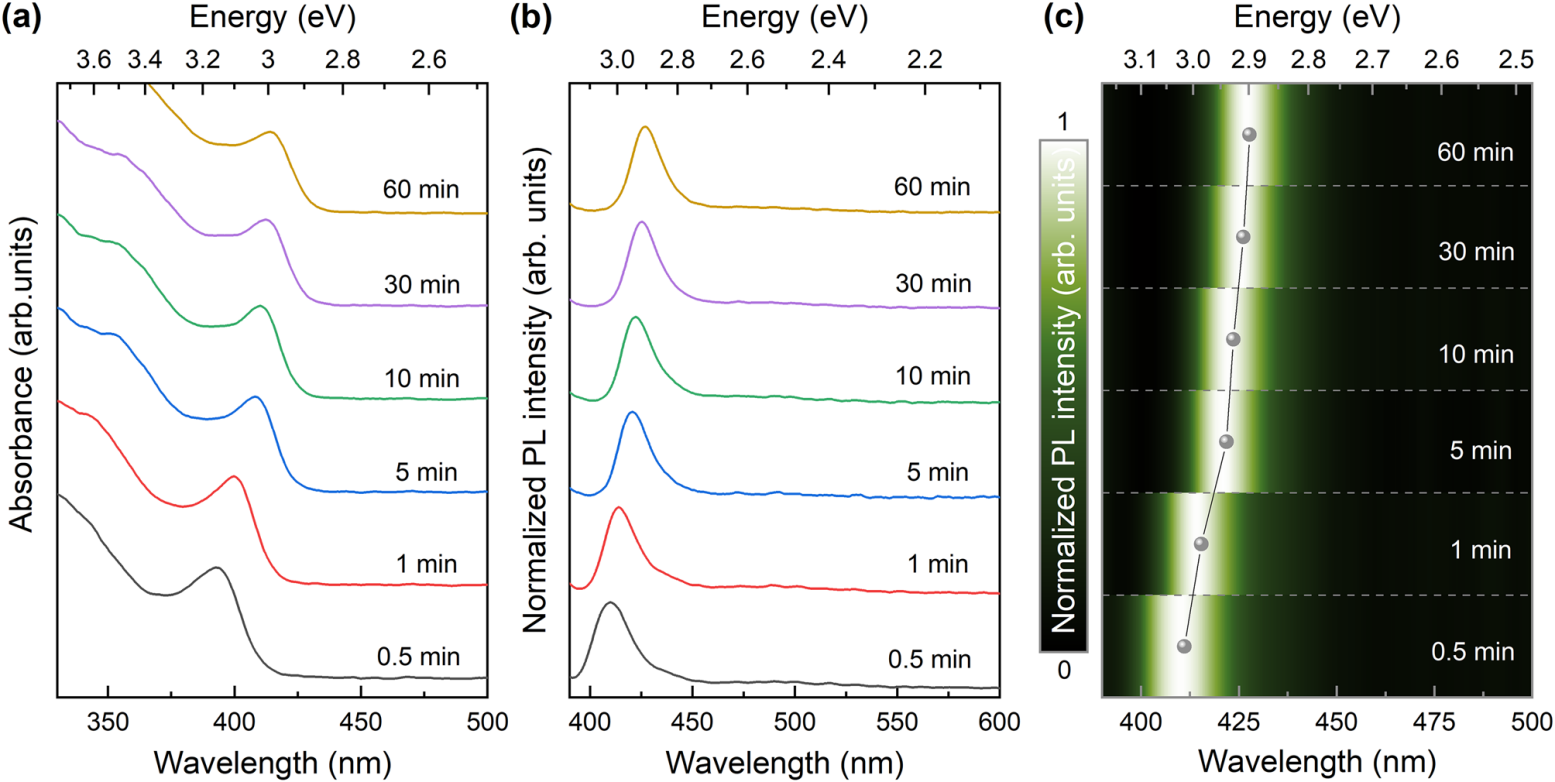
(**a**) UV–vis absorption spectra, (**b**) PL spectra, and (**c**) PL intensity of the prepared ZnSe QD samples with different reaction times.

UV–vis and PL spectroscopy are excellent tools for analyzing the optical properties of QDs, as shown in Fig. 2a, b. The absorption peaks of the prepared ZnSe QDs with reaction times of 0.5, 1, 5, 10, 30, and 60 min were observed at 393, 400, 408, 410, 412, and 414 nm, respectively. These peaks exhibit a sharp shape and well-defined excitonic features, indicating that ZnSe QDs with a narrow size distribution were formed^21,22^. The PL spectra show that the prepared ZnSe QDs fluoresce in the blue-light region, corresponding to the wavelength of their band-edge emission. No substantial defect emission is observed, although aqueous ZnSe QDs usually suffer from a broad PL band from 400 to 600 nm attributed to trapped states^23^. These results are evident in the PL intensity in Fig. 2c. The defect states, including Zn or Se vacancies, crystal defects, as well as surface states, commonly cause the light blue luminescence of ZnSe QDs^13,23–25^.

**Figure 3:**
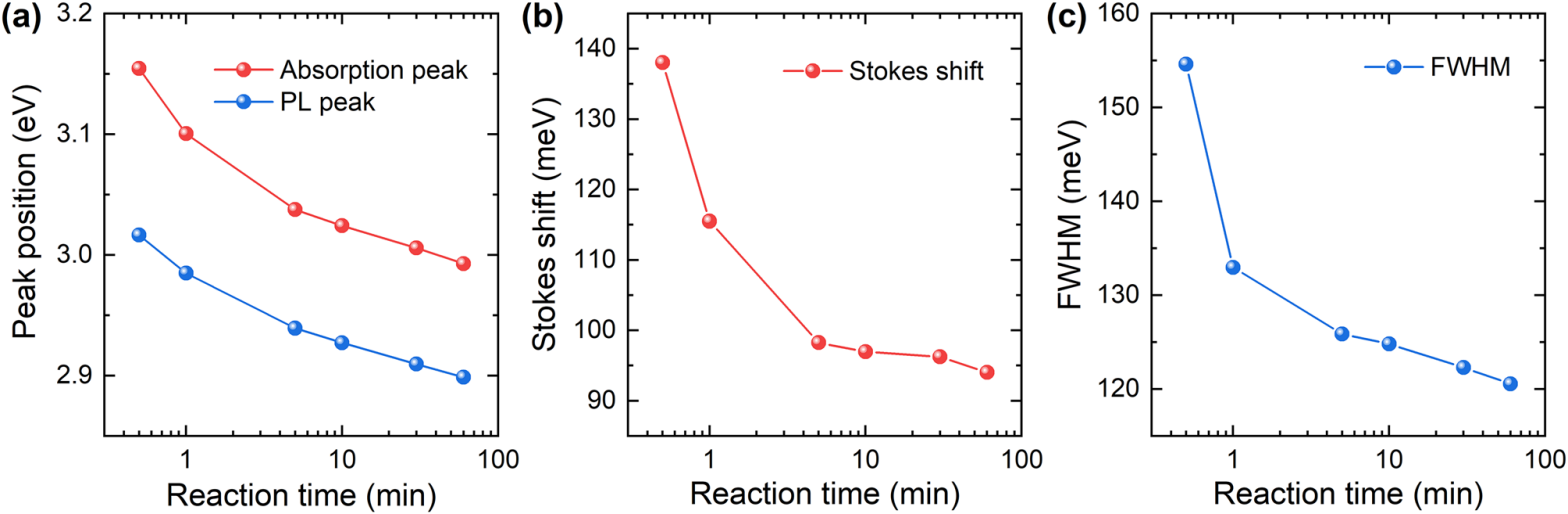
(**a**) Absorption and PL peak position in energy, (**b**) Stokes shift, and (**c**) PL FWHM of ZnSe QDs samples, plotted as functions of the reaction time.

The absorption and PL peak positions of the ZnSe QDs with different reaction times are shown in Fig. 3a. The absorption and PL peaks were observed at higher energies than the bulk ZnSe bandgap (~ 2.7 eV) due to the quantum confinement effect^9^. With increasing reaction time, the size of the ZnSe QDs increased, resulting in a red-shift of both the absorption and PL peaks. Figure 3b shows that the difference between the absorption and PL energies of the ZnSe QDs—the so-called Stokes shift—decreases from 138 to 94 meV with increasing size of the ZnSe QDs. This result indicates that the Stokes shift of the ZnSe QDs is size dependent, as observed in previous studies^26,27^.

The size distribution of the QDs has also been mentioned as a factor influencing the Stokes shift^28–30^. The bandwidth of the PL peaks is well known to be related to the size distribution of ZnSe QDs in solution^31,32^. Figure 3c shows the FWHMs of the PL peaks of the ZnSe QDs, plotted as a function of the reaction time; the FWHMs rapidly decrease within a reaction time of 5 min, indicating that the growth stages started without the formation of new nuclei. As mentioned above, burst nucleation is necessary to achieve balance between nucleation and growth; however, if either too many or too few nuclei are formed, the balance between nucleation and growth will be disrupted^15^. After the initial formation of QDs, the size distribution broadens and the sizes of the QDs in the solution tend to be larger than the critical value^15–17^, which leads to smaller QDs growing faster than the larger ones, referred to as the focusing distribution phase^33^. Both the Stokes shift and the FWHM exhibit a similar trend, showing that the correlation between the size distribution and the Stokes shift can be strong. These results from the optical properties indicate that the high quality of the prepared ZnSe QDs is attributable to the favorable balance between nucleation and growth, which results in a narrow size distribution and good surface passivation of the ZnSe QDs.

### Morphology and crystal structure analysis

**Figure 4:**
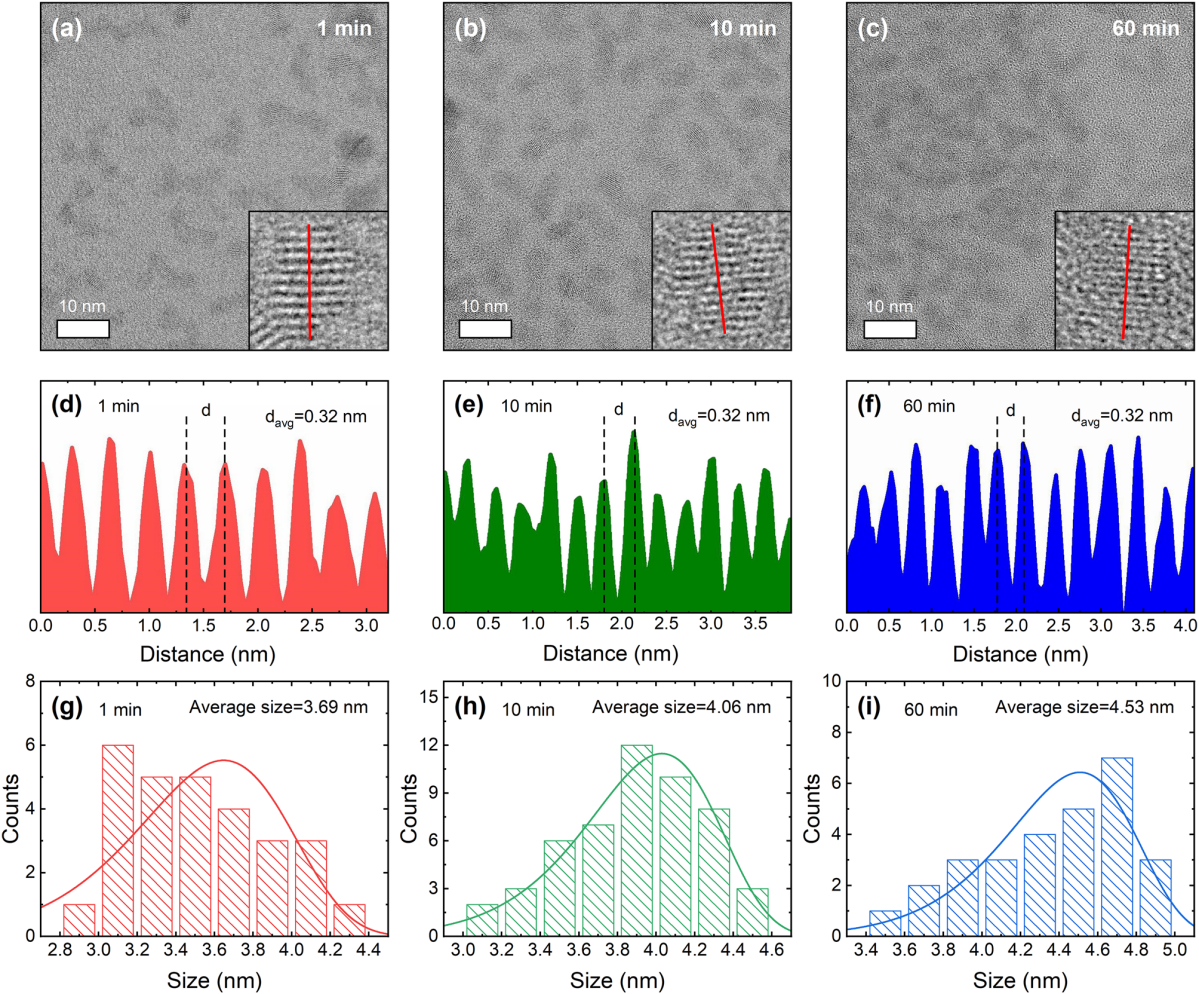
(**a**–**c**) TEM images of ZnSe QD samples collected at reaction times of 1, 10, and 60 min. The inset figures display an enlarged QD. (**d**–**f**) The enlarged QDs’ crystal profiles taken from corresponding red line in inset figures of (**a**–**c**). (**g**–**i**) The size distribution histogram with average sizes.

The morphology and crystal structure of the prepared ZnSe QDs were examined by TEM image shown in Fig. 4a–c. The samples of ZnSe QDs exhibit a spherical shape; however, the limited contrast in the TEM images poses a challenge in characterizing their dispersion. High-magnification TEM images of a particular QD clearly show lattice fringes throughout the whole QD, indicating that ZnSe QDs with highly crystalline structures were obtained^34,35^. To identify the structure type of the ZnSe QDs, the line profiles were analyzed using the Gatan Digital Micrograph software^36^ by following the line directions in the high-magnification TEM images. The interplanar spacing measurement (Fig. 4d–f) indicated an average value of 0.32 nm, which corresponds to the *d*-spacing of the (111) lattice plane of the zinc blende crystal structure^37,38^. The size of the ZnSe QDs was investigated based on TEM images using the ImageJ software^39^. The histograms showing the size distribution of the ZnSe QDs are presented in Fig. 4g–i. We determined the average QD sizes by fitting a Weibull distribution, which resulted in values of 3.69, 4.06, and 4.53 nm corresponding to growth times of 1, 10, and 60 min, respectively. These QD sizes are substantially smaller than the ZnSe Bohr diameter (~ 7.6 nm)^40,41^, which we expected because their absorption and PL peak positions are shifted to higher energies than the bulk bandgap as a result of the quantum confinement effect.

## Discussion

The optical bandgap energy (*E*_opt_) of the ZnSe QDs can be obtained from the absorption spectra and Tauc’s expression^41,42^, which is given by Eq. (1):

1 $$ \left( {\alpha h\nu } \right)^{n} = A\left( {h\nu - E_{{{\text{opt}}}} } \right) $$

where *α* is the absorption coefficient, *h* is Planck’s constant, *ν* is the photon frequency, *A* is a constant, and *n* is an index that takes the value of 2 for direct transition and 1/2 for indirect transition^41,43^. Therefore, the ZnSe QDs are a direct-bandgap material^44^. Equation (1) then becomes

2 $$ \left( {\alpha h\nu } \right)^{2} = A\left( {h\nu - E_{{{\text{opt}}}} } \right) $$

**Figure 5:**
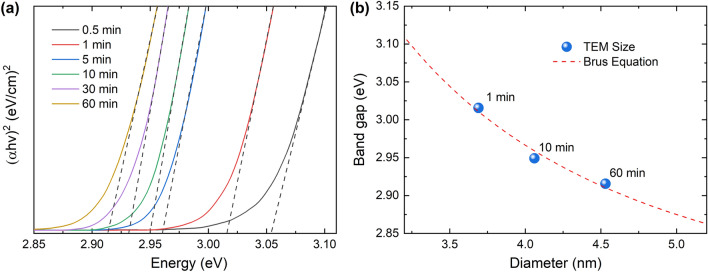
(**a**) The Tauc plot of the ZnSe QDs prepared at different reaction times. (**b**) Plot of the optical bandgap energies versus the corresponding size estimated by TEM analysis; the red dashed curve shows the fitting result for the Brus equation.

Figure 5a shows the plot of $\left( {\alpha h\nu } \right)^{2}$ as a function of the photon energy ($hv$) for the prepared ZnSe QDs. The optical bandgap energies were determined by extrapolating the linear part of the plot (dashed line) to the intersection with the *x*-axis. The obtained *E*_opt_ values are 3.05, 3.02, 2.96, 2.95, 2.93, and 2.92 eV for reaction times of 0.5, 1, 5, 10, 30, and 60 min, respectively. The optical bandgap energies decrease with increasing QD size, consistent with the quantum size effect.

Numerous theoretical models have been proposed to quantitatively correlate the expected relationship between bandgap energy (*E*_*g*_) and QD size^41^. One of the most widely used theoretical models that enables the derivation of a relatively simple analytical formula for the dependence of *E*_*g*_ on the QDs’ size is the Brus model^45^. According to this model, the bandgap of a semiconducting nanocrystal assumed to be a sphere with diameter *D* is given by

3 $$ E_{{\text{g}}} = E_{{{\text{bulk}}}} + \frac{{h^{2} }}{{2D^{2} }}\left( {\frac{1}{{m_{{\text{e}}}^{*} }} + \frac{1}{{m_{{\text{h}}}^{*} }}} \right) $$

where *E*_bulk_ is the bulk crystal bandgap value, *h* is Planck’s constant, and $m_{{\text{e}}}^{*}$ and $m_{{\text{h}}}^{*}$ are the electron and hole relative effective masses, respectively^46^. Figure 5b shows a plot of the optical bandgap energies against the corresponding size estimated by TEM analysis and the fitting curve (dashed line) based on Eq. (3). The fitting line agrees well with the analysis data of the prepared ZnSe QDs. Moreover, the *E*_bulk_ value obtained from the fitting procedure is ~ 2.71 eV, which is similar to the bandgap of bulk ZnSe. Thus, the theoretical Brus model considered here is in agreement with the experimental observation of the size dependence of ZnSe QDs on the confinement energy.

In summary, high-quality ZnSe QDs with a 1:1 ratio were successfully prepared by the hot-injection method. The resultant samples emitted blue PL with peak wavelengths ranging from 410 to 427 nm and a narrow FWHM. There was no evidence of defect emission, indicating effective surface passivation of the QDs. TEM images revealed that the synthesized ZnSe QDs had a zinc blende structure and spherical shape, with diameters ranging from 3.69 to 4.53 nm. The Brus model was used to establish a relationship between the ZnSe QD sizes and the optical bandgaps obtained from Tauc plots.

## Methods

### Materials

ZnSt_2_ (technical grade), Se powder (Se, 99.99%), 1-octadecene (ODE, technical grade, 90%), trioctylphosphine (TOP, technical grade, 90%), and ODA (≥ 99%) were purchased from Sigma-Aldrich. Chloroform (99.5%), and methanol (99.5%) were purchased from Daejung. All chemicals were used without further purification.

### Synthesis of colloidal ZnSe QDs

The synthesis of ZnSe QDs was similar to the method reported previously^10^, with some modifications. In a typical synthesis, a three-neck flask connected to a Schlenk line was loaded with a mixture of 0.19 g (0.3 mmol) ZnSt_2_ and 10 mL ODE. The mixture was evacuated at room temperature for 10 min, then heated at 90 °C for 60 min under vacuum conditions. With the mixture under flowing N_2_ gas, the temperature was then raised to 318 °C. A Se solution containing 0.04 g (0.3 mmol) Se powder, 0.15 g ODA, 0.5 mL TOP, and 1 mL ODE was rapidly injected into the mixture at 318 °C, and the temperature was maintained at 300 °C for growth of the QDs. After the injection, the colorless solution gradually turned yellow and a small aliquot was extracted from the flask at various intervals. The as-synthesized QDs were mixed with an excess of methanol, followed by centrifugation. After the upper liquid was decanted, chloroform was added to dilute the precipitate.

### Measurement techniques

The optical properties of each sample were recorded at room temperature using a FLAME-S spectrometer (Ocean Optics, Largo, FL, USA). The samples were analyzed using a TEM (JEM-ARM200F, JEOL) installed in the Center for University-wide Research Facilities at Jeonbuk National University. Samples for TEM were prepared by dipping carbon-coated TEM grids into the QD solutions and then allowing them dry under ambient air for several days. The size of the QDs was determined using ImageJ software (version 1.54g, https://imagej.net/ij/). The crystal profiles were provided using the Gatan Digital Micrograph software (version 3.6, https://www.gatan.com).

## Data Availability

The data supporting the findings of this study are available from the corresponding author upon reasonable request.
